# *Ascodipteron sanmingensis* sp. nov., a new bat fly (Hippoboscidae: streblid grade) from Fujian, China

**DOI:** 10.3897/BDJ.9.e64558

**Published:** 2021-04-23

**Authors:** Haoran Sun, Liang Ding, Liping Yan, Thomas Pape, Dong Zhang

**Affiliations:** 1 School of Ecology and Nature Conservation, Beijing Forestry University, Beijing, China School of Ecology and Nature Conservation, Beijing Forestry University Beijing China; 2 Go with KIDS Natural History Workshop, Beijing, China Go with KIDS Natural History Workshop Beijing China; 3 Natural History Museum of Denmark, Copenhagen, Denmark Natural History Museum of Denmark Copenhagen Denmark

**Keywords:** Ascodipterinae, dealate neosomic female, endoparasite, Great Himalayan Leaf-nosed Bat

## Abstract

**Background:**

The bat fly genus *Ascodipteron* Adensamer, 1896 currently contains 15 species, all of which occur in tropical and subtropical areas of the Eastern Hemisphere. A new species of endoparasitic bat fly, *Ascodipteron sanmingensis* sp. nov., was collected from the Great Himalayan Leaf-nosed Bat, *Hipposideros armiger* (Hodgson, 1853), during ecological studies on bats in Fujian, China.

**New information:**

A new species, *Ascodipteron sanmingensis* sp. nov., is described, based on dealate neosomic females and is supported by molecular data from a 368 bp fragment of the cytochrome B (Cytb) gene. Habitus and diagnostic details, as well as the attachment sites on the host, are documented with photographs. A detailed comparison of the new species with related species is provided and the new species is accommodated in the most recent key to the world species of *Ascodipteron*.

## Introduction

Bats are parasitised by some 500 species of hippoboscoid bat flies (Dick and Patterson 2006). These have traditionally been classified in the families Streblidae and Nycteribiidae, but increasing phylogenetic evidence suggests that the Streblidae are paraphyletic, with the monophyletic Nycteribiidae (which is the older name) nested inside an Old World 'streblid grade' (Petersen et al. 2007, Dittmar et al. 2015, Dittmar et al. 2006, Pape et al. 2011). Within this streblid grade, the Ascodipterine bat flies are highly specialised endoparasites, easily distinguishable from other streblids by their unusual mode of parasitic life and strong polymorphism in the adult stage, where females lose halteres, wings and legs at the trochanter immediately after attachment to a host bat and transform into neosomes (Hastriter and Bush 2006, Hastriter 2007). Apart from the monotypic genus *Maabella* Hastriter & Bush, 2006, all other ascodipterines are assigned to the genus *Ascodipteron* Adensamer, 1896 and the distribution of diagnostic features would appear to support that the two taxa most likely are sister taxa (Hastriter and Bush 2006, Hastriter 2007).

Early taxonomists, such as Adensamer (1896), Muir (1912), Monticelli (1898), Speiser (1908), Jobling (1939), Jobling (1940), Jobling (1952), Jobling (1956), Jobling (1958) and Lavoipierre (1946), studied *Ascodipteron* spp. from the Afrotropical Region, which formed the basis for further research on this genus and Maa (1965) revised the African species for the first time. Hastriter and Bush (2006) erected the genus *Maabella* and suggested *Paraascodipteron* Advani and Vazirani, 1981 should be placed in a different subfamily. Following these advances, Hastriter (2007) presented a worldwide species list, a key to all Ascodipterinae and a taxonomic review of the Oriental and Australasian species including what is known on their biology.

During the course of examining parasites collected from bats in Fujian, China, we found dealate females (neosomes) of one further undescribed species of *Ascodipteron*, which is morphologically similar to *A. phyllorhinae* Adensamer, 1896 and *A. speiserianum* Muir, 1912. Despite the flood of molecular data in the phylogenomic era (Misof et al. 2014, Kutty et al. 2019, Yan et al. 2020), morphological characters are still essential in phylogenetic and evolutionary studies (Giribet 2015). Therefore, the purpose of our study is to describe the new species and report on the host species and site of attachment and compare the new species with its most similar congeners, which will extend our knowledge of the taxonomy, distribution and biology of these parasitic flies.

## Materials and methods

### Sample collection

During October and November 2020, 10 bat flies were collected from a colony of *Hipposideros armiger*（Hodgson, 1853) roosting in an abandoned bomb shelter in a residential area of Qunying Second Village, Meilie District, Sanming, Fujian, China (26°16’30.33’’N, 117°36’49.02’’E; 195 m a.s.l.).

Entire female ascodipterine bat flies (neosomes) were removed with forceps without hurting the host. Three specimens were preserved in 75% ethanol and seven in 95% ethanol, all deposited at the Museum of Beijing Forestry University, Beijing, China (MBFU).

### Specimen imaging, measurements and terminology

Z-stack photographs were acquired with a Zeiss Axio Zoom.V16 microscope (Carl Zeiss AG, Oberkochen, Germany) equipped with a PlanApo Z 1.0×/0.25 FWD 60 objective and an AxioCam 503 colour camera. Images were processed with the software Zen 2 and Adobe Photoshop CS6 (Adobe Systems Incorporated, San Jose, USA) by cropping, contrast enhancement and removal of the background.

Ecological photographs were taken with EF 100 mm f/2.8L IS USM and MP-E 65 mm f/2.8 1-5X lenses attached to a Canon 5D Mark IV SLR camera. Images and plates were processed on a standard Windows 10 platform using Adobe Photoshop CS6 (Adobe Systems, Inc., San Jose, CA, USA).

Measurements and terminology follow Hastriter and Bush (2006).

### DNA extraction, amplification, sequencing and sequence editing

One specimen (BFU-2227) of *Ascodipteron sanmingensis* sp. nov. was selected for DNA extraction. The specimen was dissected and abdominal muscle tissue was used to extract the total genomic DNA, using the DNeasy Blood & Tissue kit (Qiagen, Dusseldorf, Germany). The remaining body parts were retained as vouchers and deposited in the entomological collection of Beijing Forestry University. A fragment of 368 bp of the cytochrome B (Cytb) gene was ampliﬁed using the primer pairs A5 (forward: 5’-AGG RCA AAT ATC ATT TTG AG-3’) and B1.1 (reverse: 5’-AAA TAT CAT TCT GGT TGA ATA TG-3’) (Dittmar and Whiting 2003). PCR reactions were conducted as described in Zhang et al. (2016) and Yan et al. (2019) and amplification conditions as described by Dittmar and Whiting (2003). The PCR products were purified and sequenced bidirectionally by BGI Inc., Beijing, China.

SeqMan Pro v. 7.1.0 (DNASTAR Inc., USA) was used to edit and assemble the forward and reverse sequences.

### DNA sequence analysis

We downloaded the only two mitochondrial cytochrome b gene (Cytb) sequences of the genus *Ascodipteron* from GenBank. The sequences, together with the Cytb sequenced in this study, were aligned using Muscle as implemented in Mega X (Kimura 1980, Kumar et al. 2018). Subsequently, nucleotide sequence divergences were calculated, using Kimura 2-parameter (K2P) model in Mega X.

## Taxon treatments

### Ascodipteron sanmingensis sp. n.

D8F0A29A-C1F4-58E9-AC17-54FF6B615201

7CB73EAD-4A34-4357-93A8-302A0B5E4462

#### Materials

- **Type status:** Holotype. **Occurrence:** recordedBy: Liang Ding; individualID: BFU-2230; individualCount: 1; sex: female; lifeStage: adult; **Taxon:** scientificName: Ascodipteron sanmingensis; phylum: Arthropoda; class: Insecta; order: Diptera; family: Streblidae; genus: Ascodipteron; specificEpithet: sanmingensis; **Location:** continent: South China; country: China; countryCode: CN; stateProvince: Fujian; county: Sanming; locality: Meilie District; verbatimLocality: Qunying Second Village; verbatimElevation: 195 m; verbatimCoordinates: 26°16’30.33’’N 117°36’49.02’’E; georeferenceProtocol: GPS; **Event:** samplingProtocol: Entire neosomes were removed with forceps from the bats; eventDate: Oct-2020; **Record Level:** language: en; institutionID: the Museum of Beijing Forestry University, Beijing, China; institutionCode: MBFU; collectionCode: Insects; basisOfRecord: PreservedSpecimen
- **Type status:** Paratype. **Occurrence:** recordedBy: Liang Ding; individualID: BFU-2225, BFU-2226, BFU-2227, BFU-2228, BFU-2229,; individualCount: 5; sex: female; lifeStage: adult; **Taxon:** scientificName: Ascodipteron sanmingensis; phylum: Arthropoda; class: Insecta; order: Diptera; family: Streblidae; genus: Ascodipteron; specificEpithet: sanmingensis; **Location:** continent: South China; country: China; countryCode: CN; stateProvince: Fujian; county: Sanming; locality: Meilie District; verbatimLocality: Qunying Second Village; verbatimElevation: 195 m; verbatimCoordinates: 26°16’30.33’’N 117°36’49.02’’E; georeferenceProtocol: GPS; **Identification:** dateIdentified: 2020; **Event:** samplingProtocol: Entire neosomes were removed with forceps from the bats; eventDate: Oct-2020; **Record Level:** language: en; institutionID: the Museum of Beijing Forestry University, Beijing, China; institutionCode: MBFU; collectionCode: Insects; basisOfRecord: PreservedSpecimen
- **Type status:** Paratype. **Occurrence:** recordedBy: Liang Ding & Hao-Ran Sun; individualID: BFU-2221, BFU-2222; individualCount: 2; sex: female; lifeStage: adult; **Taxon:** scientificName: Ascodipteron sanmingensis; phylum: Arthropoda; class: Insecta; order: Diptera; family: Streblidae; genus: Ascodipteron; specificEpithet: sanmingensis; **Location:** continent: South China; country: China; countryCode: CN; stateProvince: Fujian; county: Sanming; locality: Meilie District; verbatimLocality: Qunying Second Village; verbatimElevation: 195 m; verbatimCoordinates: 26°16’30.33’’N 117°36’49.02’’E; georeferenceProtocol: GPS; **Identification:** dateIdentified: 2020; **Event:** samplingProtocol: Entire neosomes were removed with forceps from the bats; eventDate: 11/15/2020; **Record Level:** language: en; institutionID: the Museum of Beijing Forestry University, Beijing, China; institutionCode: MBFU; collectionCode: Insects; basisOfRecord: PreservedSpecimen
- **Type status:** Paratype. **Occurrence:** recordedBy: Liang Ding & Hao-Ran Sun; individualID: BFU-2223, BFU-2224; individualCount: 2; sex: female; lifeStage: adult; **Taxon:** scientificName: Ascodipteron sanmingensis; phylum: Arthropoda; class: Insecta; order: Diptera; family: Streblidae; genus: Ascodipteron; specificEpithet: sanmingensis; **Location:** continent: South China; country: China; countryCode: CN; stateProvince: Fujian; county: Sanming; locality: Meilie District; verbatimLocality: Qunying Second Village; verbatimElevation: 195 m; verbatimCoordinates: 26°16’30.33’’N 117°36’49.02’’E; georeferenceProtocol: GPS; **Identification:** dateIdentified: 2020; **Event:** samplingProtocol: Entire neosomes were removed with forceps from the bats; eventDate: 11/17/2020; **Record Level:** language: en; institutionID: the Museum of Beijing Forestry University, Beijing, China; institutionCode: MBFU; collectionCode: Insects; basisOfRecord: PreservedSpecimen

#### Description

**Female. Head**. Labial theca slightly longer than wide (Fig. 2B,D; Fig. 3B,C); posterior margin concave dorsally, convex ventrally. Labial theca dorsally with ca. 50＋ pigmented, peg-like, spiniform setae and ventrally with ca. 100＋ similar setae. Peg-like setae identical to those on gena. Gena with ca. 40 peg-like setae on dorsal half; anterior margin convex (Fig. 3A). Posterior margin of frons trilobed. Arista with multiple fine branches; basal antennal segment with a single long seta. Lateral vertex with convex antero-medial margin; adorned with 30–36 long setae; about twice as long as wide, with a longitudinal fold in the lateral portion of the sclerite (Fig. 4A). Occipital sclerite triangular (Fig. 2B).

**Thorax**(Fig. 2B,D; Fig. 3A; Fig. 4B). Scutum with numerous long setae, especially numerous along posterolateral margin; devoid of setae along mid-line. Mesopleuron with 8–10 short, peg-like spiniform setae anterior to large round spiracle; setae posterior to spiracle of three varieties: 6–7 short, peg-like, spiniform setae (ventral); 6–8 longer, sharp, spiniform setae (medial); 8–10 long, slender setae (along posterior margin and dorsal). Pteropleuron with 20–26 short, peg-like, spiniform setae in dorsal half of sclerite; ventral half devoid of setae. Hypopleuron and sternopleuron without setae. Coxa 1 with 12–15 peg-like, spiniform setae and 3 long, slender setae on proximal half. Coxae 2 and 3 each with a cluster of 8–11 long, slender setae. Trochanters 1 and 2 each with 2–3 minute, spiniform setae on anterior apex; 1–2 slender setae on posteroapical margin. Prosternum devoid of setae, mesosternum with 2–3 slender setae, metasternum with 4 short setae and posterolateral margin distinctly concave.

**Genital Aperture** (Fig. 4C and D). Five terminal annular rings arranged roughly into rows comprised of different types of setae (abbreviated R1–5, with R1 the proximal and R5 the distal row). R1, R2 and R4 with minute, thick setae; R3 and R5 with long setae. VSS (ventral spiracular setae situated in a ventral, arching row or grouping between spiracles #7) of 19–23 long setae. MSS (medial spiracular setae) arranged in symmetrical groups of 2–3 setae, situated between spiracles #6 and #7. DSS (dorsal spiracular setae) arranged in a single dorsal arching row of 7–8 long setae between spiracles #5. Cercus with 4–6 setae, diameter ca. 43 μm.

**Dimensions.** Head and thorax: 1671 μm (n = 3, range: 1666–1676 μm); Labial theca, length: 626 μm (n = 2, range: 556–695 μm); width: 511 μm (n = 2, range: 495–526 μm), genital aperture, diameter: 1171 μm (n = 2, range: 1125–1217 μm), neosome, length: 4655 μm.

**Male.** Unknown.

#### Diagnosis

Labial theca slightly longer than wide (Fig. 2B and D; Fig. 3B and C), R1, R2, R4 as minute, thick setae and R3, R5 as long setae. Most similar to *A. phyllorhinae and A. speiserianum*, but separable from the former by having 50＋ spiniform setae dorsally on the labial theca and a lateral fold on the lateral vertex and from the latter by the setose lateral vertex (Fig. 4A).

#### Etymology

The new species is named after its type locality Sanming.

#### Distribution

Oriental – China (Fujian).

#### Biology

**Figure 1.**
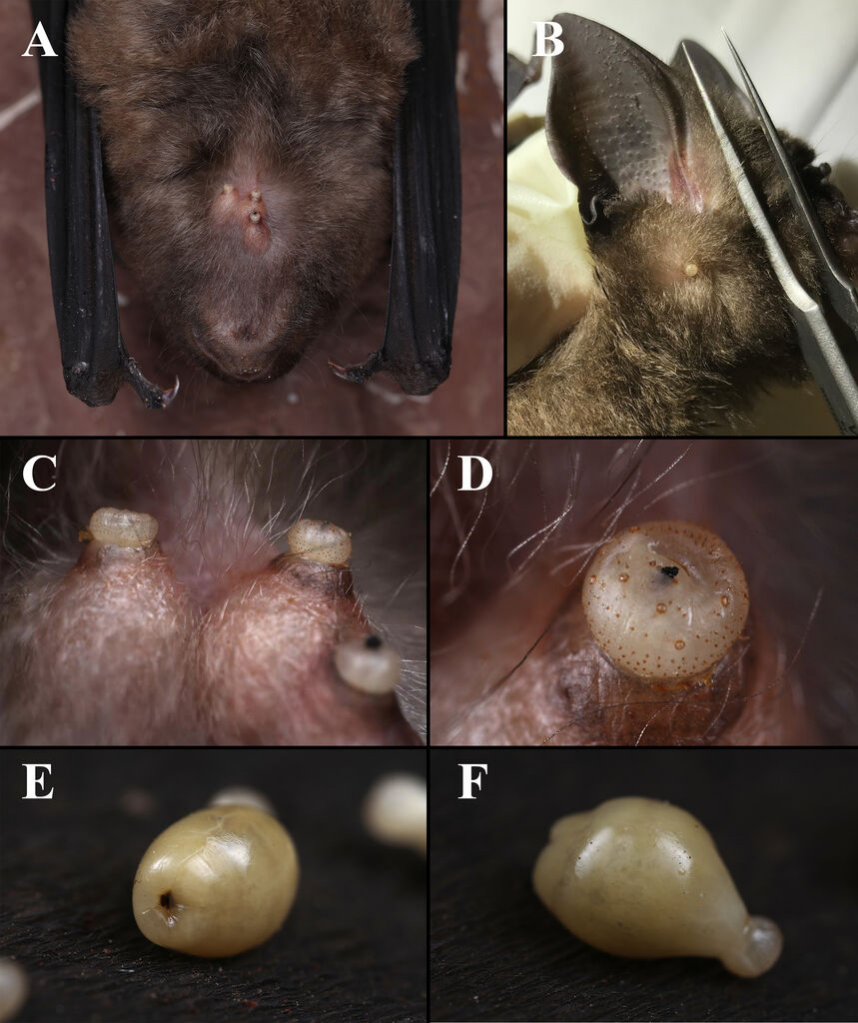
*Ascodipteron sanmingensis* sp. nov. and its host *Hipposideros armiger*. **A.** Neosomes protruding from the lower jaw area of host. **B.** Neosomes protruding from the base of an ear of host. **C–D.** Neosomes embedded in host tissue. **E–F.** Whole neosomes freshly extracted from host (head and thorax fully withdrawn).

**Figure 2.**
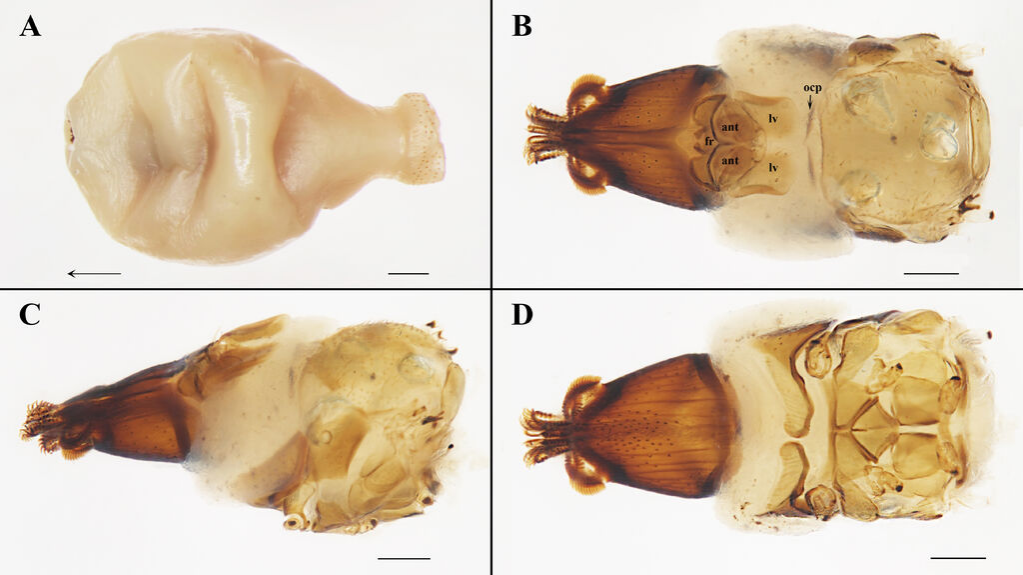
*Ascodipteron sanmingensis* sp. nov., ex. *H. armiger*, China. **A.** Whole neosome (head and thorax fully withdrawn, the arrow indicates the direction of the head) (BFU–2227, paratype). **B–D.** Head and thorax (BFU–2228, paratype); dorsal view (B), lateral view (C) and ventral view (D). Abbreviations: ant, antenna; fr, frons; lv, lateral vertex; and ocp, occipital sclerite. Scale bars: A = 500 μm; B–C = 200 μm.

**Figure 3.**
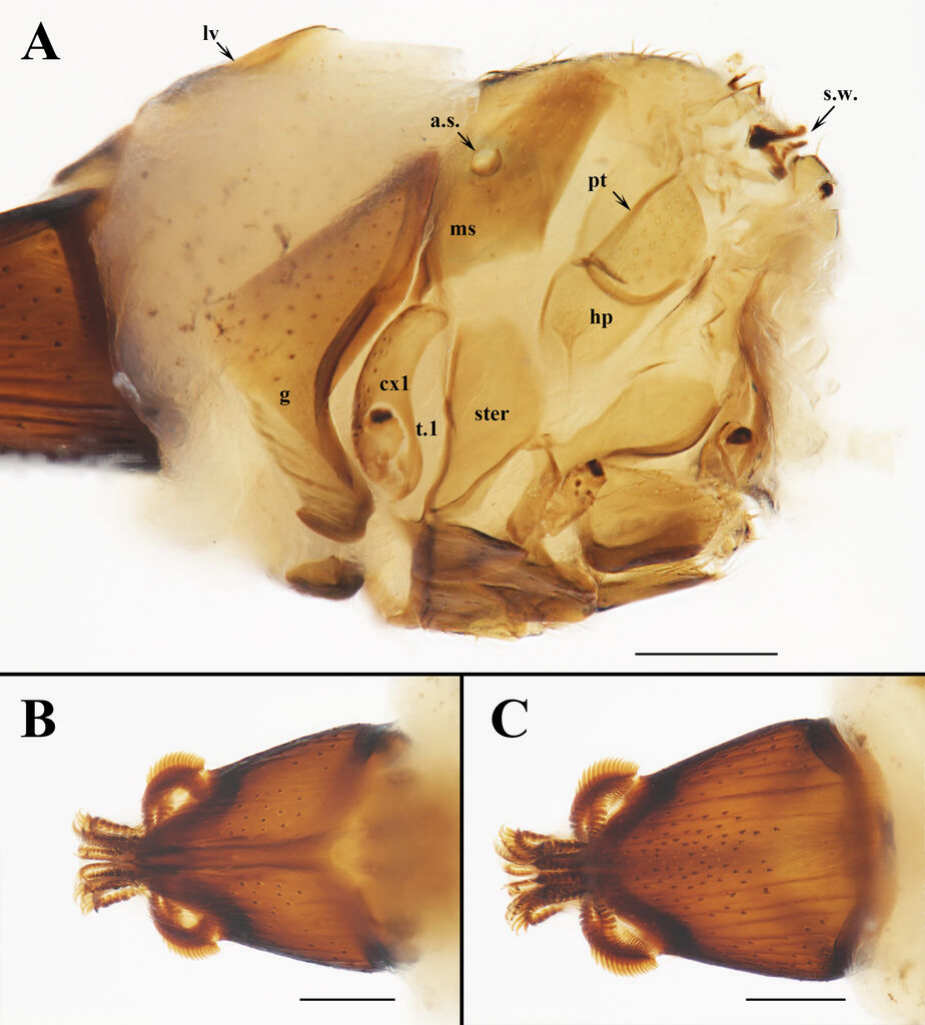
*Ascodipteron sanmingensis* sp. nov., ex. *H. armiger*, China (BFU–2228, paratype). **A.** Thorax, lateral dorsal view. **B.** Labial theca, dorsal view. **C.** Labial theca, ventral view. Abbreviations: a.s., anterior thoracic spiracle; cx1, coxa 1; g, gena; hp, hypopleuron; lv, lateral vertex; ms, mesopleuron; pt, pteropleuron; s.w., stump of wing; ster, sternopleuron; and t.1, trochanter 1. Scale bars: A–C = 200 μm.

**Figure 4.**
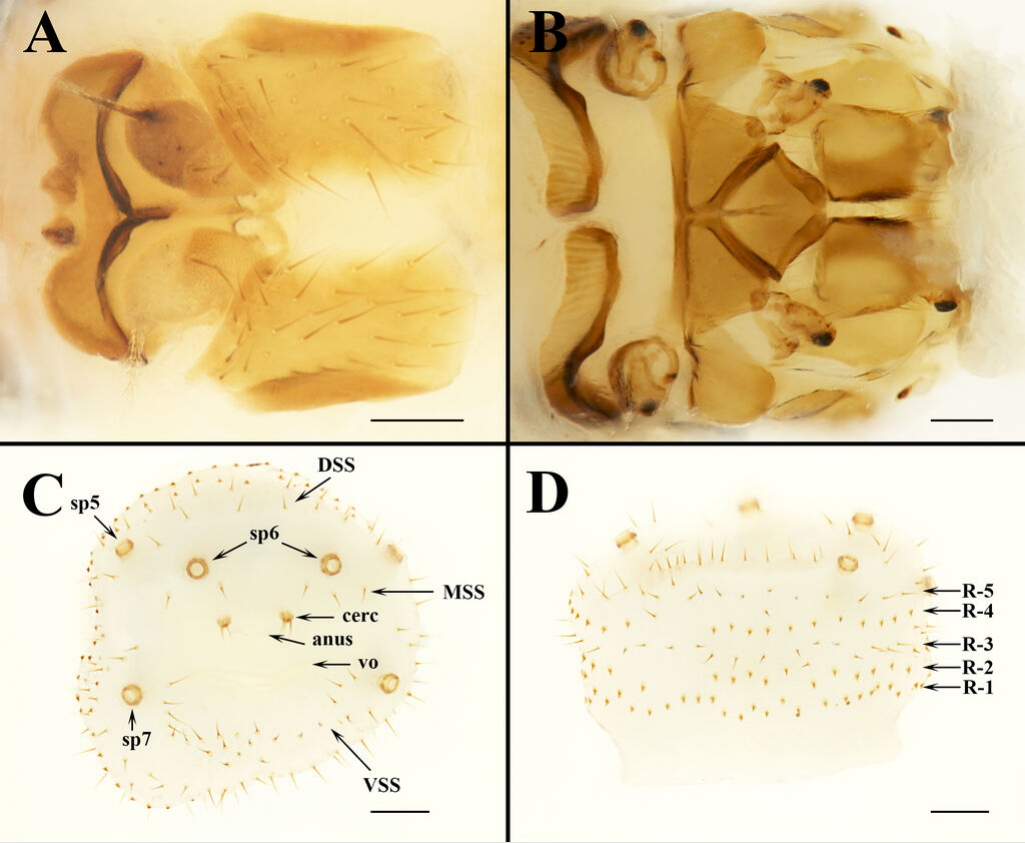
*Ascodipteron sanmingensis* sp. nov., ex. *H. armiger*, China. **A.** Frons and lateral vertex (BFU–2221, paratype). **B.** Thorax, ventral view (BFU–2221, paratype). **C.** Abdomen, posterior view (BFU–2223, paratype). **D.** Five terminal annular rows of setae (BFU–2223, paratype). Abbreviations: sp5–sp7, spiracles 5–7; DSS, dorsal spiracular setae; MSS, medial spiracular setae; VSS, ventral spiracular setae; anus, anus; cerc, cercus; vo, vaginal orifice; R1–5, abdominal setae arranged roughly into annular rows comprised of variable types of setae, R1 the proximal and R5 the distal row. Scale bars: A–B = 100 μm; C–D = 200 μm.

*Ascodipteron sanmingensis* sp. nov. is embedded at the base of an ear or on the lower jaw area of *Hipposideros armiger* (Fig. 1A–D). The neosomes are not inserted perpendicular to the skin, but at a slight angle.

#### Molecular results

The genetic distance between *Ascodipteron sanmingensis* sp. nov. and *A. phyllorhinae* is 7.75% and between *A. sanmingensis* sp. nov. and an unidentified *A.* sp. is 11.86% (Table 1). The Cytb sequence generated in this study was deposited in GenBank (Accession: MW822598).

## Identification Keys

### Remarks:

**Table:** 

1	Medial spiracular setae (MSS) comprised of two or three setae. First abdominal annular row (R1) present.	2
–	Medial spiracular setae (MSS) comprised of two setae grouped adjacent to spiracle #6. First abdominal annular row (R1) absent. [*Rhinolophus*, Africa.]	*A. brevior*
2	Labial theca dorsally with 25–30 lightly pigmented peg-like spiniform setae. Lateral vertex without fold or reinforcement in the lateral portion of the sclerite. [*Hipposideros* spp., usually on wing, SE China to Solomon Islands.]	*A. phyllorhinae*
–	Labial theca dorsally with ca. 50＋ pigmented peg-like spiniform setae. Lateral vertex with a longitudinal fold or reinforcement in the lateral portion of the sclerite. [*Hipposideros armiger*, at the base of ear or on the lower jaw area, only known from Fujian, China.]	*A. sanmingensis* sp. nov.

*Ascodipteron sanmingensis* sp. nov. will run to couplet 14 in the identification key to dealate ascodipterine females proposed by Hastriter (2007) and it can be incorporated in the key as follows, with host data given in square brackets:

## Discussion

Hastriter (2007) revised the species of *Ascodipteron* from the Oriental and Australasian regions and provided a detailed review of what is known of their biology. He studied extensive material of *A. phyllorhinae* from across its extensive range and he mentioned that the specimens "may prove to represent more than one species" (p. 13). Given the very small morphological differences between *A. phyllorhinae* and *A. sanmingensis* sp. nov., a closer examination of the lectotype of *A. emballoneurae* Banks from Borneo would seem justified.

Only two other species of *Ascodipteron* are, so far, known to use *Hipposideros armiger* as host: *A. longiascus* Hastriter, 2007, known in four specimens obtained from a single specimen of *H. armiger* in China (Yunnan) and *A. phyllorhinae*, which appears to favour *H. diadema* Geoffroy, 1813 as its host (Hastriter 2007).

*Ascodipteron sanmingensis* sp. nov. is only known from China (Fujian), although its known host has a much wider distribution over most of South-East Asia (Bates et al. 2020). Hastriter (2007) concluded that "Of the hundreds of specimens studied across the entire Oriental and Australasian regions … there are only two previously described sympatric *Ascodipteron* species, *A. phyllorhinae* and *A. speiserianum*". Both *A. phyllorhinae* and *A. speiserianum* have broad distributional ranges, but are, in general, of a more southern distribution, with *A. phyllorhinae* reaching north into southern China (Guangxi) and *A. speiserianum* reaching further north into China (Hainan, Taiwan) and southern Japan (Hastriter 2007). Further sampling is needed to fully assess the degree of sympatry for these three species.

Liu et al. 2018 evaluated the potential use of Cytb for insect species identification and found it almost as efficient as COI with a success rate of 95% correct identifications. (Gilarriortua et al. 2013, Liu et al. 2018).

Dittmar et al. 2006 provided molecular data from two species of *Ascodipteron* from a single locality in Malaysia, Pahang. These were identified as *A. phyllorhinae* and a species given as new to science, but not described. Comparing data from the present material with Cytb sequences provided by Dittmar et al. 2006 reveals a difference of 7.75% with their *A. phyllorhinae* and of 11.86% with the species considered to be undescribed (Table 1). The intraspecific variation of Cytb for insects has been estimated to be 0.66 ± 0.81% by Liu et al. (2018) in able 2, indicating that *A. sanmingensis* sp. nov. is a different species from *A. phyllorhinae* and *A.* sp.

## Supplementary Material

XML Treatment for Ascodipteron sanmingensis

## Figures and Tables

**Table 1.** Pairwise differences of mitochondrial cytochrome b gene (Cytb) sequences between species, based on Kimura 2-parameter

		1	2
1	*Ascodipteron sanmingensis* sp. nov. (MW822598)		
2	*Ascodipteron phyllorhinae* (DQ133149.1)	0.0775	
3	*Ascodipteron* sp. (DQ133154.1)	0.1186	0.1057
